# Tomato leaf disease recognition based on multi-task distillation learning

**DOI:** 10.3389/fpls.2023.1330527

**Published:** 2024-01-30

**Authors:** Bo Liu, Shusen Wei, Fan Zhang, Nawei Guo, Hongyu Fan, Wei Yao

**Affiliations:** ^1^ College of Information Science and Technology, Hebei Agricultural University, Baoding, China; ^2^ Hebei Key Laboratory of Agricultural Big Data, Baoding, China

**Keywords:** multi-task learning, knowledge distillation, tomato leaf diseases, disease classification, severity prediction

## Abstract

**Introduction:**

Tomato leaf diseases can cause major yield and quality losses. Computer vision techniques for automated disease recognition show promise but face challenges like symptom variations, limited labeled data, and model complexity.

**Methods:**

Prior works explored hand-crafted and deep learning features for tomato disease classification and multi-task severity prediction, but did not sufficiently exploit the shared and unique knowledge between these tasks. We present a novel multi-task distillation learning (MTDL) framework for comprehensive diagnosis of tomato leaf diseases. It employs knowledge disentanglement, mutual learning, and knowledge integration through a multi-stage strategy to leverage the complementary nature of classification and severity prediction.

**Results:**

Experiments show our framework improves performance while reducing model complexity. The MTDL-optimized EfficientNet outperforms single-task ResNet101 in classification accuracy by 0.68% and severity estimation by 1.52%, using only 9.46% of its parameters.

**Discussion:**

The findings demonstrate the practical potential of our framework for intelligent agriculture applications.

## 1 Introduction

Tomato is one of the most widely cultivated vegetables in the world, with its versatility extending to various applications such as a culinary ingredient (Kumar et al., 2022), an industrial raw material (Botineştean et al., 2015), a component in cosmetics (Septiyanti and Meliana, 2020), and medicinal uses (Kumar et al., 2012). However, tomato diseases can rapidly spread through a field if not identified and managed in a timely manner, leading to substantial losses in both yield and quality of the crop (Zhang et al., 2022). As symptoms of many tomato diseases can appear on the leaves, leveraging computer vision techniques for automated recognition of leaf diseases has attracted widespread attention from researchers (Boulent et al., 2019; Habib et al., 2020; Nanehkaran et al., 2020; Roy and Bhaduri, 2021; Albahli and Nawaz, 2022; Harakannanavar et al., 2022). Although these techniques effectively improve the accuracy and speed of disease diagnosis, they also present challenges. These include variations in disease symptoms and lighting conditions (Zhang et al., 2018a), difficulty in collecting enough disease samples (Zhang et al., 2021), varying levels of disease severity (Wang et al., 2021), and limitations in computing power (Bi et al., 2022). Such factors potentially influence the applicability of the learning models.

Most of the computer vision-based leaf disease recognition methods are mainly divided into two categories: hand-crafted feature-based methods and deep learning-based methods. Traditionally, hand-crafted features refer to the manual extraction of specific features such as textures, colors, shapes, and sizes from leaf images. These features are then used as input for training a classifier to identify the presence of plant diseases. The utilization of classical classifiers, such as support vector machines (SVM) (Cortes and Vapnik, 1995) and random forests (RF) (Breiman, 2001), has been instrumental in leaf disease identification, owing to their robust nature in handling high-dimensional, noisy, and missing data (Patil et al., 2017). Consequently, the research community has significantly focused on developing improved methods for feature extraction to enhance recognition performance. Mokhtar et al. (2015) employed geometric features and histogram features for classifying two tomato leaf viruses, achieving the highest accuracy of 91.5% using the Quadratic kernel function. Meenakshi et al. (2019) improved plant leaf disease identification using exact Legendre moments shape descriptors, with a high accuracy of 99.1% on three tomato diseases (early and late blight and mosaic). In Rahman et al. (2022), texture features from tomato leaf images were analyzed using a gray level co-occurrence matrix (GLCM). In addition to single-type features, hybrid features have been well-studied. Sharif et al. (2018) proposed a hybrid method for automatic detection and classification of six types of diseases in citrus plants, which used color, texture, and geometric features combined in a codebook and selected by PCA score, entropy, and skewness-based covariance vector before being fed to a multi-class SVM. Similarly, Basavaiah and Arlene Anthony (2020) recognized four main diseases in tomato plants through the fusion of multiple features, including color histograms, Hu Moments, Haralick, and local binary pattern, resulting in 94% accuracy achieved by a RF classifier. In summary, hand-crafted feature-based methods are highly valued for their simplicity and interpretability, as well as they have demonstrated satisfactory performance on small to medium-sized datasets. However, they struggle to scale up large and diverse datasets, and fall short in coping with biases and noises in the data distribution, leading to decreased accuracy and robustness in real-world applications.

Recently, deep learning has revolutionized the field of computer vision, resulting in significant improvements in detecting leaf diseases (Sujatha et al., 2021; Shoaib et al., 2022). For instance, a novel tomato leaf disease recognition framework was proposed, which used binary Wavelet transform for image preprocessing to remove noise, and both-channel residual attention network (B-ARNet) for identification (Sujatha et al., 2021). Other types of attention mechanisms are also incorporated to enhance the model’s recognition capability. In Zhao et al. (2021), to adaptively recalibrate channel-wise feature responses, a squeeze-and-excitation (SE) module (Hu et al., 2018) is integrated into a ResNet50 network (He et al., 2016), with an average identification accuracy of 96.81% on the publicly available PlantVillage dataset (Hughes et al., 2015).

Additionally, Bhujel et al. (2022) compared the performance and computational complexity of different attention modules and found that the convolutional block attention module (CBAM) (Woo et al., 2018) was the most effective in enhancing classification performance, resulting in an average accuracy of 99.69%. Despite the successes of these deep learning-based methods, they face limitations such as the need for large amounts of labeled data and substantial computational resources. To address these challenges, researchers have proposed a series of strategies for constructing lightweight networks, such as depthwise separable convolutions (MobileNet (Howard et al., 2017)), channel shuffling (ShuffleNet (Zhang et al., 2018a)), and a combination of network scaling and architecture search (EfficientNet (Tan and Le, 2019)). For example, Zeng et al. (2022) developed a lightweight CNN model named LDSNet, which uses an improved dense dilated convolution (IDDC) block and coordinated attention scale fusion (CASF) mechanism to identify corn leaf diseases in complex backgrounds. Similarly, Janarthan et al. (2022) utilized a simplified MobileNetV2 architecture and an empirical method for creating class prototypes, requiring low processing power and storage space. Li et al. (2023) explored a hybrid transformer-based architecture by integrating shuffle-convolution and a lightweight transformer encoder. While compact models achieve computational efficiency gains by reducing the parameters, these gains may come at the cost of decreased accuracy (Atila et al., 2021; Thai et al., 2023).

In addition to identifying the presence of a plant disease, it is also crucial to estimate the severity of the disease, providing a quantitative assessment for disease diagnosis (Ilyas et al., 2022; Ji and Wu, 2022). The precise localization, size, and distribution of infected regions in plant leaves can significantly enhance the accuracy of disease classification, especially in field images with complex backgrounds (Barbedo, 2019). Moreover, these factors are vital for severity grading, disease progression monitoring, and assessment of treatment efficacy. The process of estimating the level of leaf diseases often involves two main steps: segmentation and grading. Segmentation refers to the operation of separating infected regions from healthy areas of the leaf or plant. This can be achieved through various methods such as morphological operations (Gupta, 2022), k-means clustering and thresholding (Karlekar and Seal, 2020; Singh et al., 2021), and deep learning-based semantic segmentation (Wang et al., 2021; Liu et al., 2022; Deng et al., 2023). Grading then assigns a numerical score or rating to the severity of the disease, based on proportional area measurement (Wu et al., 2022) or ordinal categories (Ozguven and Adem, 2019; Pal and Kumar, 2023). Considering the complementary nature of disease classification and severity estimation, there is an emerging trend toward multi-task learning. This approach aims to jointly optimize both tasks by leveraging shared representations and correlations between them. For example, Ji et al. (2020) presented a set of binary relevance-CNNs that can simultaneously recognize 7 crop species, classify 10 crop diseases (including healthy), and estimate 3 disease severity levels, achieving the best test accuracy of 86.70% for recognition and 92.93% for severity estimation. Other techniques, such as alternating training (Jiang et al., 2021) and weighting adjustment (Wang et al., 2022), have been explored to enhance the accuracy of the combined task. Although multi-task learning can lead to better performance than individual tasks, it may also introduce increased computational effort and suboptimal solutions due to the difficulty in balancing tasks.

To address these challenges, we propose a novel multi-task distillation learning framework for tomato leaf disease diagnosis (MTDL). Unlike traditional distillation learning (Hinton et al., 2015) that relies on one-to-one and one-way knowledge transfer from a teacher model to a student model. Instead, our framework considers tomato disease category identification and severity prediction as a multi-task model that can be optimized simultaneously, as well as two single-task models that can be mutually informative. Accordingly, we develop a learning process for knowledge decoupling and reorganization, facilitating the efficient transfer of knowledge between the two tasks. Furthermore, this process is designed to be integrated with deep networks of varying complexity and architecture, making it adaptable to different disease identification scenarios with diverse computational power configurations and performance requirements.

Specifically, MTDL uses a multi-task model that contains disease classification and severity estimation as the baseline. It adopts a multi-stage learning strategy, including knowledge disentanglement, single-task mutual learning, and knowledge integration, In this process, the goal of knowledge disentanglement is to transfer the shared knowledge from the original multi-task model to the corresponding single-task models. This enables the specialization of task-specific models and avoids negative transfer of knowledge between tasks. For mutual learning between tasks, the goal is to fully exploit the complementarity between different learning objectives. Finally, through knowledge integration, the disentangled and mutually learned knowledge components are re-combined and unified to produce the refined high-quality multi-task model.

Furthermore, considering that multi-stage distillation learning will lead to a dependency of the current student model on the teacher model from the previous stage, we propose a decoupled teacher-free knowledge distillation (DTF-KD) strategy to simplify the training process. DTF-KD introduces a virtual teacher, replacing the traditional teacher model in the distillation process. This approach allows for increased adaptability by applying different learning intensities to target and non-target knowledge. In the context of the classification problem addressed in this paper, the target knowledge corresponds to the correct classification assignment of the ground truth.

The main contributions of this paper are summarized as follows:

1. We propose a novel multi-task distillation learning (MTDL) framework for leaf disease identification. This framework progressively decomposes and integrates the inherent knowledge from two tasks: tomato disease classification and severity prediction, through a distillation process, thereby generating a robust multi-task model for comprehensive disease diagnosis.
2. We propose a decoupled teacher-free knowledge distillation (DTF-KD) method to simplify MTDL by reducing the reliance on teacher models during the learning process. A virtual teacher is introduced to guide the learning process by providing separate instructions for the correct class and non-correct classes.
3. The experimental results demonstrate that the proposed framework effectively leverages the complementary characteristics of tomato disease category identification and severity prediction, reducing the model size while improving the performance.

## 2 Materials and methods

### 2.1 Dataset

**Figure 1:**
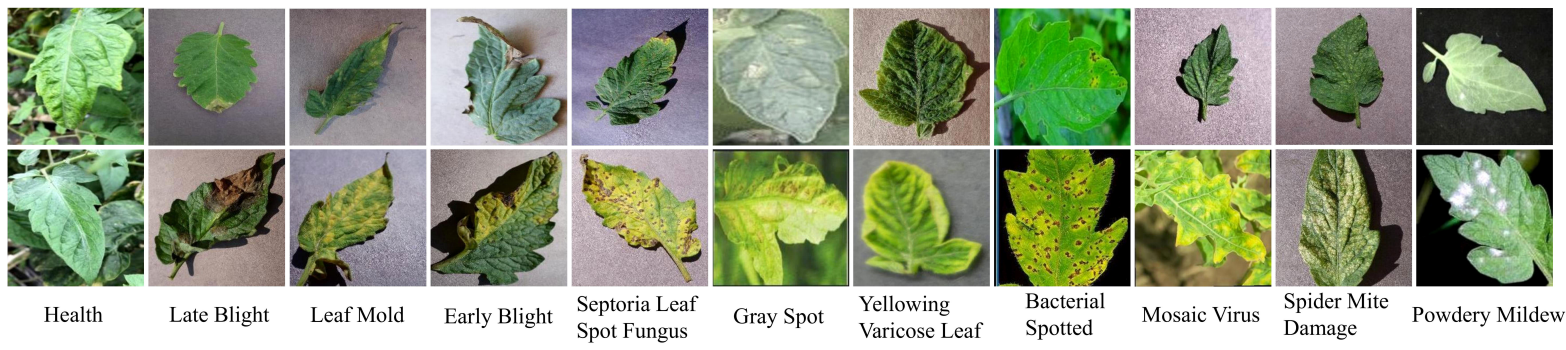
Examples of tomato diseases from the datasets.

The dataset employed in this study is aggregated from three distinct sources.The first source is drawn from the AI Challenger 2018 Crop Leaf Disease Challenge (Dataset AI Challenger, 2018), encompassing 11 types of plants and 27 types of diseases. Some of these diseases are further categorized into general and severe degrees, resulting in a total of 61 categories. Specifically, the dataset includes instances of leaf diseases for the following plants: apple (2,765), grape (3,144), peach (2,146), potato (3,246), citrus (4,577), pepper (1,929), strawberry (1,263), cherry (939), maize (3,514), pumpkin (1,465), and tomato (11,610). For the purposes of our study, we focus on the tomato subset. However, as the dataset contains only three samples of Canker disease, we decide to exclude this category from our analysis. The second source, the PlantDoc dataset (Singh et al., 2020), consists of 2,598 data samples that involve 13 types of plants and 27 categories (17 diseases, 11 healthy). These samples were mainly obtained from the internet and manually annotated, with the tomato subset containing 8 categories. The third source is the Taiwan Tomato Disease dataset (Huang and Chang, 2020), which is originally comprising 622 samples, was first employed in the study detailed in Thuseethan et al. (2022). In addition, it encompasses six distinct categories, namely Bacterial Spotted (110), Leaf Mold (67), Gray Spot (84), Health (106), Late Blight (98), and Powdery Mildew (157). We choose this dataset for its diverse disease conditions and combine it with larger datasets like AI Challenger 2018 and PlantDoc to further enrich the diversity of our data. **Figure 1** shows examples of different tomato leaf diseases.

### 2.2 Data preprocessing

For the AI Challenger dataset, given the scarcity of data for the canker disease category (only 3 instances), we excluded this data. The dataset provided severity labels for most of the data, categorized into three levels: healthy, moderate, and severe. In addition, we supplemented the dataset with severity labels for the tomato spotted wilt virus. For the PlantDoc dataset, due to the complexity of the leaf background, we manually cropped the tomato leaf subset to meet the needs of the disease identification task. Each image was cropped to retain the main area of a single leaf while preserving some background information from the plant. For the Taiwan Tomato dataset, we used all the original data. For all three datasets, we applied consistent severity labeling. Specifically, we hired five agricultural experts to manually annotate the severity of the disease. The final severity level was determined by a majority vote. **Table 1** summarizes the information about the three datasets used in this study.

**Table 1:** Summary of main datasets used in the study.

Dataset	AIChallenger2018			PlantDoc			Taiwan			Total
Class	Healthy	Moderate	Severe	Healthy	Moderate	Severe	Healthy	Moderate	Severe	
Health	1381			120			106			1607
Late Blight		302	1267		10	29		16	82	1706
Leaf Mold		371	384		40	67		22	45	929
Early Blight		287	505		22	86				900
Septoria Leaf Spot Fungus		481	922		23	141				1567
Gray Spot								25	59	84
Yellowing Varicose Leaf		1616	2790		35	88				4529
Bacterial Spotted		47	27		15	56		29	81	255
Mosaic Virus		104	194		26	43				367
Spider Mite Damage		619	310							929
Powdery Mildew								47	110	157
Total	1381	3827	6399	120	171	510	106	139	377	13030

We divide the dataset into training, validation, and test sets in an 8:1:1 ratio, ensuring a balanced and representative distribution for each set. The division is performed randomly to maintain fairness and diversity. Furthermore, we rigorously validate both the results reported in the paper and the determination of hyperparameters through 10-fold cross-validation.

### 2.3 Multi-task distillation framework

**Figure 2:**
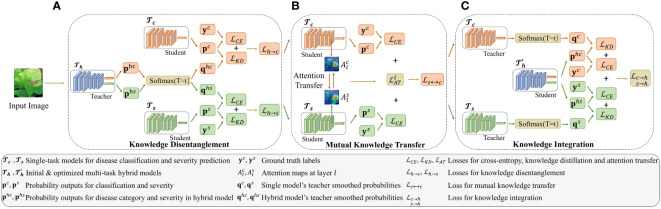
Architecture of the multi-task distillation learning (MTDL). The MTDL framework uses a three-stage distillation process involving single-task models $T_{c}$ and $T_{s}$ , and a multi-task model $T_{\text{h}}$ . Initially, knowledge from $T_{\text{h}}$ is transferred to the single-task models. Then, $T_{c}$ and $T_{s}$ share knowledge. Finally, their knowledge is integrated back into $T_{\text{h}}$ , creating an improved multi-task model $T_{h}^{'}$ . For simplicity, sample indices are omitted from the symbols in the figure. Additionally, the temperature parameter *T* in KD is fixed at *t* during the learning process. **(A)** Knowledge Disentanglement, **(B)** Mutual Knowledge Transfer, **(C)** Knowledge Integration.

The proposed MTDL for tomato leaf disease diagnosis is comprised of three components: two single-task models, one for disease recognition and the other for severity prediction, and a hybrid model that integrates these two tasks. As illustrated in **Figure 2**, the MTDL pipeline enables mutual knowledge transfer between the two individual tasks, facilitating knowledge disentanglement and integration to enhance performance. In traditional distillation learning processes (Hinton et al., 2015), a powerful teacher model transfer knowledge to a lightweight student model. However, our MTDL framework emphasizes bidirectional knowledge transfer between teacher and student models, allowing for greater flexibility in their selection.

#### 2.3.1 Problem formulation

Given a leaf disease dataset $D={\left\{({\text{x}}_{i},y_{i}^{c},y_{i}^{s})\right\}}_{i=1}^{N}$ containing *N* images, where $x_{i}\in {\mathbb{R}}^{C\times H\times W}$ is the *i*-th leaf image with *C*, *H*, and *W* denoting the number of channels, height, and width of the image, respectively. Each image is labeled with two types of annotations: $y_{i}^{c}\in \{1,2,\cdot \cdot \cdot ,K^{c}$ is the disease category label, with *K^c^* being the number of disease categories, and $y_{i}^{s}\in \{1,2,\cdot \cdot \cdot ,K^{s}\}$ is the disease degree label, with *K^s^* being the number of severity levels.

In MTDL, there are three basic tasks denoted as $T_{c}$ for disease category recognition, $T_{s}$ for severity estimation, and $T_{h}$ for the hybrid task that jointly performs $T_{c}$ and $T_{s}$ . As shown in **Figure 2**, each task uses a standard ResNet50 (He et al., 2016) as the backbone for feature extraction. In particular, the two single tasks $T_{c}$ and $T_{s}$ , each uses a multi-layer perceptron (MLP) to output the logits of its corresponding task, denoted as ${\text{z}}_{i}^{c}\in \;{\mathbb{R}}^{K_{c}}$ and ${\text{z}}_{i}^{s}\in \;{\mathbb{R}}^{K_{s}}$ , respectively. For $T_{h}$ , two separate MLPs are used to perform two tasks simultaneously on a shared backbone, and the output is denoted as ${\text{z}}_{i}^{h}=\left[{\text{z}}_{i}^{hc}:{\text{z}}_{i}^{hs}\right]\in \;{\mathbb{R}}^{K_{c}+K_{s}}$ , where ${\text{z}}_{i}^{hc}$ and ${\text{z}}_{i}^{hs}$ corresponding to the logits for the disease category and severity, respectively. Usually, a softmax function is applied to the output of each task to produce the predicted probabilities, ${\text{p}}_{i}^{c}\in \;{\mathbb{R}}^{K^{c}}$ , ${\text{p}}_{i}^{s}\in \;{\mathbb{R}}^{K^{s}}$ and ${\text{p}}_{i}^{h}=\left[{\text{p}}_{i}^{hc}:p_{i}^{hs}\right]\in \;{\mathbb{R}}^{K_{c}+K_{s}}$ , respectively. Guided by these three basic tasks, MTDL employs a designed knowledge routing mechanism to build a tomato disease diagnosis model. The process begins with the distillation of multi-task knowledge from $T_{h}$ back to the corresponding task models $T_{c}$ and $T_{s}$ (as shown in **Figure 2A**). These two models then engage in mutual learning (as shown in **Figure 2B**). Finally, the knowledge from these two models is integrated to output an enhanced multi-task model, namely $T^{\prime }{\;}_{h}$ (as shown in **Figure 2C**). The detailed learning process is described in the following sections, including, knowledge decomposition (Section 2.3.2), mutual knowledge tranfer (Section 2.3.3), and knowledge integration (Section 2.3.4).

#### 2.3.2 Knowledge disentanglement

Multi-task learning has demonstrated its advantages in leveraging shared information among related tasks to improve performance on individual tasks. However, directly training a multi-tasking model can be suboptimal, as the tasks may have different levels of difficulty. For instance, the task of severity estimation is more challenging than the leaf disease classification task because it typically necessitates a finer analysis of the leaf and disease spot attributes (Wang et al., 2017). Therefore, given a multi-task model $T_{h}$ pre-trained on dataset *D*, as shown in **Figure 2A**, it is reasonable to disentangle the shared knowledge and transfer it back to the single-task models, i.e., $T_{c}$ and $T_{s}$ , using knowledge distillation (Hinton et al., 2015). Specifically, when distilling knowledge from $T_{h}$ to $T_{c}$ , we first soften the probability ${\text{p}}_{i}^{hc}$ by:


(1) $$q_{i,j}^{hc}=\frac{\exp\;\left(p_{i,j}^{hc}/T\right)}{\sum_{j}\text{exp }\left(p_{i,j}^{hc}/T\right)}$$

where *T* is the temperature hyperparameter that controls the sharpness of ${\text{q}}_{i}^{hc}$ , $p_{i,j}^{hc}$ is the *j*-th element of ${\text{p}}_{i}^{hc}$ , and $q_{i,j}^{hc}$ denotes the softened probability distribution of the *j*-th class for the *i*-th input data. The formulation of the knowledge distillation process from $T_{h}$ to $T_{c}$ involves minimizing the loss function ${ℒ}_{h\to c}$ , which is defined as follows:


(2) $${ℒ}_{h\to c}=\frac{1}{N}\sum_{i=1}^{N}\left[{ℒ}_{CE}\left({\text{p}}_{i}^{c},{\text{y}}_{i}^{c}\right)+{ℒ}_{KD}\left({\text{p}}_{i}^{c},{\text{q}}_{i}^{hc}\right)\right]$$

where $\;{ℒ}_{CE}$ is the cross-entropy loss, which measures the dissimilarity between the predicted probability distribution ${\text{p}}_{i}^{c}$ and the one-hot ground-truth label vector ${\text{y}}_{i}^{c}$ for the single-task model $T_{c}$ . It can be written as shown in Equation 3:


(3) $${ℒ}_{CE}\left({\text{p}}_{i}^{c},{\text{y}}_{i}^{c}\right)=-\sum_{j=1}^{K_{c}}y_{i,j}^{c}\text{log }p_{i,j}^{c}$$

And ${ℒ}_{KD}$ , the knowledge distillation loss, which quantifies the divergence between $q_{i}^{hc}$ and $p_{i}^{c}$ , is defined as shown in Equation 4:


(4) $${ℒ}_{KD}\left({\text{p}}_{i}^{c},{\text{q}}_{i}^{hc}\right)=\sum_{j=1}^{K_{c}}q_{i,j}^{hc}\text{log }\frac{q_{i,j}^{hc}}{p_{i,j}^{c}}$$

Similar to Equation 2, we can define a loss function from

$T_{h}$ to $T_{s}$ , denoted as ${ℒ}_{h\to s}$ , which is given by:


(5) $${ℒ}_{h\to s}=\frac{1}{N}\sum_{i=1}^{N}\left[{ℒ}_{CE}\left({\text{p}}_{i}^{s},{\text{y}}_{i}^{s}\right)+{ℒ}_{KD}\left({\text{p}}_{i}^{s},{\text{q}}_{i}^{hs}\right)\right]$$

where ${\text{q}}_{i}^{hs}$ is the probability distribution obtained by softening the severity prediction output ${\text{p}}_{i}^{hs}$ from $T_{h}$ (referred to in Equation 1), and ${\text{p}}_{i}^{s}$ is the output from $T_{s}$ .

#### 2.3.3 Mutual knowledge transfer

Upon completing the knowledge disentanglement process, the shared knowledge from the hybrid tasks $T_{h}$ is individually transferred back to the corresponding subtasks, i.e., $T_{c}$ for disease species classification and $T_{s}$ for disease severity identification. We then employ mutual distillation to further investigate the complementarity of the two subtasks. Here, we assume that $T_{c}$ and $T_{s}$ use the same backbones, such as ResNet50. Motivated by Komodakis and Zagoruyko (2016), as shown in **Figure 2B**, the commonality of knowledge between subtasks is reflected in the consistency of attention maps from the middle layer. Specifically, given two feature mappings, $F_{l}^{c}$ and $F_{l}^{s}$ , which are the outputs of layer *l* of the models $T_{c}$ and $T_{s}$ , respectively, we can calculate the attention maps, $A_{l}^{c}$ and $A_{l}^{s}$ , as shown in Equation 6:


(6) $$A_{l}^{c}\left(x,y\right)=\frac{1}{C_{i}}\sum_{c=1}^{C_{i}}F_{l}^{c}\left(k,x,y\right),\;A_{l}^{s}\left(x,y\right)=\frac{1}{C_{i}}\sum_{k=1}^{C_{i}}F_{l}^{s}\left(k,x,y\right)$$

where *C_i_* is the number of channels in the feature mappings of

$F_{l}^{c}$ and $F_{l}^{s}$ , and (*k,x,y*) specifies the location and channel of an activation value within the feature mapping. The attention maps $A_{l}^{c}$ and $A_{l}^{s}$ are computed by averaging the activation values across the channels of the respective feature mappings, $F_{l}^{c}$ and $F_{l}^{s}$ . For stability of optimization, we first reshape the $A_{l}^{c}$ and $A_{l}^{s}$ into a vector form as ${\text{a}}_{l}^{c}=\text{vec}\left(A_{l}^{c}\right)$ and ${\text{a}}_{l}^{s}=\text{vec}\left(A_{l}^{s}\right)$ , where vec(.) is an operation that transforms a matrix into a vector by concatenating its columns. Then, we normalize the vectors using *l*_2_ norm as shown in Equation 7:


(7) $${\hat{a}}_{l}^{c}=\frac{a_{l}^{c}}{\|a_{l}^{c}\|{\;}_{2}},\;{\hat{a}}_{l}^{s}=\frac{a_{l}^{s}}{\|a_{l}^{s}\|{\;}_{2}}$$

The attention transfer loss for layer *l* is written as shown in Equation 8:


(8) $${ℒ}_{AT}\left({\hat{a}}_{l}^{c},{\hat{a}}_{l}^{s}\right)=\|{\hat{a}}_{l}^{c}-{\hat{a}}_{l}^{s}\|{\;}_{2}^{2}$$

And the total loss for mutual learning between subtasks is defined as follows:


(9) $${ℒ}_{s↔c}=\frac{1}{N}\sum_{i=1}^{N}\left[{ℒ}_{CE}\left({\text{p}}_{i}^{c},{\text{y}}_{i}^{c}\right)+{ℒ}_{CE}\left({\text{p}}_{i}^{s},{\text{y}}_{i}^{s}\right)\right]+\frac{1}{L}\sum_{l=1}^{L}{ℒ}_{AT}\left({\hat{\text{a}}}_{l}^{c},{\hat{\text{a}}}_{l}^{s}\right)]$$

where *L* denotes the number of layers considered for attention transfer loss.

#### 2.3.4 Knowledge integration

The primary objective of the proposed MTDL is to enhance multi-task learning capabilities. In the final step of this learning framework, we consider the two sub-tasks after mutual learning, $T_{c}$ and $T_{s}$ , and reintegrate them into the original multi-tasking model, denoted as … As shown in **Figure 2C**, this reintegration process results in an enhanced multi-task model $T_{h}^{'}$ .The knowledge integration loss is formulated as follows:


(10) $${ℒ}_{\begin{array}{c} c\to h\\ s\to h \end{array}}=\frac{1}{N}\sum_{i=1}^{N}[{ℒ}_{CE}({\text{p}}_{i}^{hc},{\text{y}}_{i}^{c})+{ℒ}_{KD}({\text{p}}_{i}^{hc},{\text{q}}_{i}^{c})+{ℒ}_{CE}({\text{p}}_{i}^{hs},{\text{y}}_{i}^{s})+{ℒ}_{KD}({\text{p}}_{i}^{hs},{\text{q}}_{i}^{s})]$$

where ${\text{q}}_{i}^{c}$ and ${\text{q}}_{i}^{s}$ represent the output of softened probability distributions of $T_{c}$ and $T_{s}$ , respectively, which are obtained by applying the process described in Equation 1. The whole process of MTDL is summarized in **Algorithm 1**.

**Algorithm 1:**
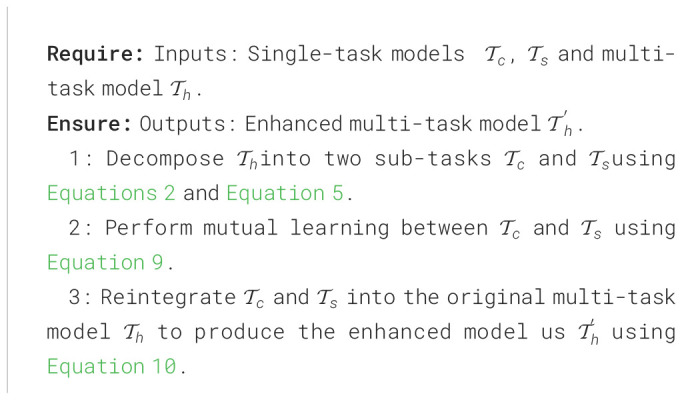
MTDL process.

### 2.4 Teacher-free based MTDL

In the staged learning process of MTDL, the current stage can be considered the teacher model for subsequent stages. While this approach fully utilizes the process of knowledge transfer, it also leads to a dependency on the teacher model, thereby reducing the flexibility of the framework. To overcome this limitation, inspired by the work of Yuan et al. (2020) and Zhao et al. (2022), we propose a decoupled teacher-free KD (DTF-KD) method. In the following sections, we first present the general form of the DTF-KD, and then demonstrate how it can be applied to MTDL.

In the absence of a teacher model, we introduce a virtual teacher. We define the output of this virtual teacher as a categorical distribution, *v_i,j_*, given by:


(11) $$v_{i,j}=\{\begin{array}{ll} \alpha & \text{ if }j=t\\ \left(1-\alpha \right)/\left(K-1\right) & \text{ if }j\in \backslash t \end{array}$$

where *α* is a predefined constant, typically ≥ 0.95, *t* is the correct class or target class for the *i*-th sample, *K* is the total number of classes, *j* represents the class index, and \*t* denotes all classes except the correct class *t*. This definition ensures that the virtual teacher assigns the highest probability to the correct class, while distributing the remaining probability equally among the incorrect classes.

In our proposed DTF-KD method, we divide the information distillation process into two parts: teacherfree based correct class KD (CC-KD) and teacher-free based non-correct class KD (NCC-KD). CC-KD focuses on the learning of target knowledge. It aims to transfer knowledge that is particularly important or challenging for the student model. In CC-KD, according to Equation 11, the binary probability outputs the virtual teacher for the correct class *t* and the *K*−1 non-correct classes are denoted as ${\text{q}}_{i}^{v}=\;\left[q_{i,t}^{v},\;q_{i,\backslash t}^{v}\right]\;\in \;{\mathbb{R}}^{2}$ . These outputs are calculated using:


(12) $$q_{i,t}^{v}=\frac{\text{exp}\;(\alpha )}{\text{exp}\;(\alpha )+\sum_{k=1,k\neq t}^{K}\text{exp}\;\left(v_{i,k}\right)},\;q_{i,\backslash t}^{v}=\frac{\sum_{k=1,k\neq t}^{K}\text{exp}\;\left(v_{i,k}\right)}{\text{exp}\;(\alpha )+\sum_{k=1,k\neq t}^{K}\text{exp}\;\left(v_{i,k}\right)}$$

Correspondingly, for the student model, we can obtain $b_{i}=\;\left[b_{i,t},b_{i,}{\;}_{\backslash t}\right]\;\in {\mathbb{R}}^{2}$ , defined as: 


(13) $$b_{i,t}=\frac{\exp\;\left(z_{i,t}\right)}{\sum_{j=1}^{K}\exp\;\left(z_{i,j}\right)},\;b_{i,\setminus t}=\frac{\sum_{k=1,k\neq t}^{K}\text{exp }\left(z_{i,k}\right)}{\sum_{j=1}^{K}\exp\;\left(z_{i,j}\right)}$$

where *z_i,j_* represents the logit for the *j*-th class of *i*-th instance of the student model. Therefore, combining Equations 12 and 13, the loss function of CC-KD can be written as:


(14) $${ℒ}_{CC-KD}\left(b_{i},q_{i}^{v}\right)=q_{i,t}^{v}\log\;\frac{q_{i,t}^{v}}{b_{i,t}}+q_{i,\setminus t}^{v}\log\;\frac{q_{i,\setminus t}^{v}}{b_{i,\setminus t}}$$

In NCC-KD, we consider the probability outputs for the *K*−1 non-correct classes, denoted as ${\tilde{\text{q}}}_{i}^{v}\in {\mathbb{R}}^{K}{\;}^{-1}$ for the virtual teacher and ${\tilde{\text{p}}}_{i}\in {\mathbb{R}}^{K}{\;}^{-1}$ for the student model. For each *m* ∈ {1, 2,…,*K*}\{*t*}, we calculate these outputs as follows:


(15) $${\tilde{q}}_{i,m}^{v}=\frac{\text{exp }(v_{i,m})}{\sum_{k=1,k\neq t}^{K}\exp\;(v_{i,k})},\;{\tilde{p}}_{i,m}=\frac{\exp\;\left(z_{i,m}\right)}{\sum_{k=1,k\neq t}^{K}\exp\;\left(z_{i,k}\right)}$$

where *v_i,m_* is defined in Equation 11, and *z_i,m_* represents the logit for the *m*-th class of the *i*-th instance from the student model. According to Equation 15, the NCC-KD loss function is then defined as:


(16) $${ℒ}_{NCC-KD}\left({\tilde{\text{p}}}_{i},{\tilde{\text{q}}}_{i}^{v}\right)=\sum_{j=1,j\neq t}^{K}{\tilde{q}}_{i,j}^{v}\log\;\frac{{\tilde{q}}_{i,j}^{v}}{{\tilde{p}}_{i,j}}$$

Combining Equations 14 and 16, the total loss of DTF-KD is


(17) $${ℒ}_{DFK-KD}\left(b_{i},q_{i}^{v},{\tilde{p}}_{i},{\tilde{q}}_{i}^{v}\right)={ℒ}_{CC-KD}\left(b_{i},q_{i}^{v}\right)+{ℒ}_{NCC-KD}\left({\tilde{p}}_{i},{\tilde{q}}_{i}^{v}\right)$$

**Figure 3:**
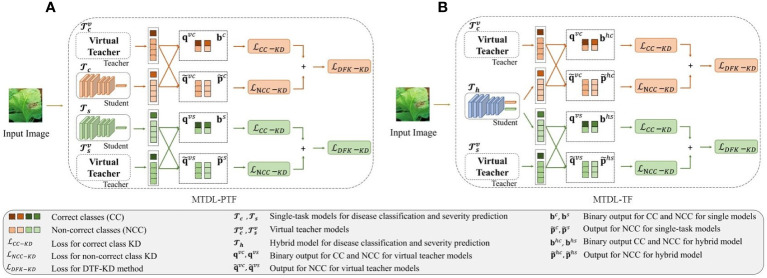
Overview of the decoupled teacher-free (DTF) based MTDL. **(A)** Partially teacher-free MTDL (MTDL-PTF): Eliminating dependency on the multi-task teacher model in the knowledge disentanglement stage. **(B)** Teacher-Free MTDL (MTDL-TF): Simplifying MTDL to only retain the final knowledge integration stage with virtual teachers.


According to DTF-KD, we propose two variants of the MTDL framework. The first variant, as shown in **Figure 3A** which we call partially teacher-free MTDL (MTDL-PTF), eliminates the knowledge disentanglement stage from the MTDL process, thereby removing the dependency on the initial multi-task teacher model, known as $T_{h}$ . To compensate for the absence of $T_{h}$ , we introduce two virtual teacher models corresponding to the two learning tasks of disease category recognition and severity estimation, denoted as $T_{c}^{v}$ and $T_{s}^{v}$ , respectively. For $T_{c}^{v}$ , as described in Equations 12, 13 and 15, we obtain ${\text{q}}_{i}^{vc}\in \;{\mathbb{R}}^{2}$ and $b_{i}^{c}\in {\mathbb{R}}^{2}$ for the distillation outputs for the correct class, as well as and ${\tilde{\text{q}}}_{i}^{vc}\in \;{\mathbb{R}}^{K^{c}-1}$ and ${\tilde{\text{p}}}_{i}^{c}\in \;{\mathbb{R}}^{K^{c}-1}$ the non-correct classes. Similarly, for $T_{s}^{v}$ , we can obtain $q_{i}^{vs}\in \;{\mathbb{R}}^{2}$ and $b_{i}^{s}\in {\mathbb{R}}^{2}$ for the correct severity level. For the non-correct severity levels, we can also obtain ${\tilde{\text{q}}}_{i}^{vs}\in \;{\mathbb{R}}^{K^{s}-1}$ and ${\tilde{p}}_{i}^{vs}\in \;{\mathbb{R}}^{K^{s}-1}$ . Therefore, the mutual knowledge transfer process in MTDL-PTF is given as shown in Equation 18:

(18) $${ℒ}_{s↔c}^{v}={ℒ}_{s↔c}+\frac{1}{N}[\sum_{i=1}^{N}{ℒ}_{DFK-KD}\left({\text{b}}_{i}^{c},{\text{q}}_{i}^{vc},{\tilde{\text{p}}}_{i}^{c},{\tilde{\text{q}}}_{i}^{vc}\right)+\sum_{i=1}^{N}{ℒ}_{DFK-KD}\left({\text{b}}_{i}^{s},{\text{q}}_{i}^{vs},{\tilde{\text{p}}}_{i}^{s},{\tilde{\text{q}}}_{i}^{vs}\right)]$$

where ${ℒ}_{s↔c}$ and ${ℒ}_{DFK-KD}$ L*_DFK_*_-_ *_KD_* are defined in Equations 9 and 17, respectively.

In the second variant of MTDL, named teacher-free MTDL (MTDL-TF), we completely abandon the teacher model. The process of MTDL-TF is illustrated in **Figure 3B**. Instead, we directly introduce the distillation information from the virtual teacher models $T_{c}^{v}$ and $T_{s}^{v}$ into $T_{h}$ , which is defined as shown in Equation 19:


(19) $${ℒ}_{\begin{array}{l} c\to h\\ s\to h \end{array}}^{v}=\frac{1}{N}\sum_{i=1}^{N}\begin{array}{c} {}[{ℒ}_{CE}\left({\text{p}}_{i}^{hc},{\text{y}}_{i}^{c}\right)+{ℒ}_{DFK-KD}({\text{b}}_{i}^{hc},{\text{q}}_{i}^{vc},{\tilde{\text{p}}}_{i}^{hc},{\tilde{\text{q}}}_{i}^{vc})\\ +{ℒ}_{CE}\left({\text{p}}_{i}^{hs},{\text{y}}_{i}^{s}\right)+{ℒ}_{DFK-KD}\left({\text{b}}_{i}^{hs},{\text{q}}_{i}^{vs},{\tilde{\text{p}}}_{i}^{hs},{\tilde{\text{q}}}_{i}^{vs}\right)] \end{array}$$

where ${\text{b}}_{i}^{hc}$ and ${\text{b}}_{i}^{hs}$ are two binary probability outputs corresponding to the correct class and non-correct classes for the disease category recognition and severity estimation tasks, respectively, in the hybrid model $T_{h}$ . They can be obtained via ${\text{z}}_{i}^{hc}$ and ${\text{z}}_{i}^{hs}$ using Equation 13. Accordingly, the output for the non-correct classes in $T_{h}$ , ${\tilde{\text{p}}}_{i}^{hc}$ and ${\tilde{\text{p}}}_{i}^{hs}$ , can be calculated by Equation 15.

## 3 Experimental results and discussion

### 3.1 Experimental setup

#### 3.1.1 Model training

The MTDL framework consists of three main components: knowledge disentanglement, subtask mutual learning, and knowledge integration. To ensure simplicity and generality of the framework, we employ a consistent training strategy for different learning components. Specifically, the framework is trained using the SGD optimizer with a batch size of 32 and a momentum of 0.9. The initial learning rate is set to 0.001, and it is reduced by a factor of 0.1 every 20 epochs. The weight decay is set to 1e-4. The maximum number of training epochs is set to 100, and an early stopping strategy is used based on the validation performance. If the validation loss does not improve for 5 consecutive epochs, the training process is stopped.

#### 3.1.2 Hyperparameter settings

The MTDL framework involves three main stages of knowledge distillation, which correspond to the objective functions in Equations 2, 9, and 10. During the process, we use a temperature parameter *T* to smooth the output of the teacher model. This hyperparameter is determined through cross-validation using the validation set. A comprehensive analysis of hyperparameter selection can be found in Section 3.3.4.

#### 3.1.3 Evaluation metrics

To evaluate the performance of the proposed MTDL method, we employ four commonly used evaluation metrics, namely Accuracy, Precision, Recall, and F1-score. Given true positives (TP), true negatives (TN), false positives (FP), and false negatives (FN), the specific definitions of these metrics are as shown in Equations 20 and 21:


(20) $$\text{Accuracy}=\frac{\text{TP}+\text{TN}}{\text{TP}+\text{FP}+\text{FN}+\text{TN}},\text{ Precision}=\frac{\text{TP}}{\text{TP}+\text{FP}},\text{ Recall}=\frac{\text{TP}}{\text{TP}+\text{FN}}$$

(21) $$\text{F}1-\text{score}=2\times \frac{\text{Precision}\times \text{Recall}}{\text{Precision}+\text{Recall}}$$

#### 3.1.4 Baseline methods

The MTDL framework is a flexible knowledge distillation approach designed for tomato disease diagnosis. It aims to improve the performance of recognition models while reducing their parameter size and can be combined with various existing neural network architectures. To ensure the versatility of the MTDL framework, we incorporate four conventional network models, including ResNet101 (He et al., 2016), ResNet50 (He et al., 2016), DenseNet121 (Huang et al., 2017), and VGG16 (Simonyan and Zisserman, 2014), as well as four lightweight network models such as EfficientNet (Tan and Le, 2019), ShuffleNetV2 (Zhang et al., 2018b), MobileNetV3 (Howard et al., 2019), and SqueezeNet (Iandola et al., 2016). Detailed information about these models can be found in **Table 2**. These backbone models serve as the learning components in different stages of the MTDL framework. We use the original classification results of these models as a baseline and compare the results before and after the multi-task distillation process to validate the effectiveness of the proposed framework.

**Table 2:** Baseline results of single and multi-task models.

Methods	Single Task (Accuracy)		Multi Task (Accuracy)		Single Task (F1-score)		Multi Task (F1-score)		Parameter	FLOPs
	$\;T_{c}$	$T_{s}$	$T_{hc}$	$T_{hs}$	$\;T_{c}$	$T_{s}$	$T_{hc}$	$T_{hs}$	(M)	(G)
VGG16	96.68	93.34	96.76 (↑0.08)	93.43 (↑0.09)	96.57	94.34	96.82 (↑0.25)	94.53 (↑0.19)	253.864	15.699
ResNet101	98.11	93.61	98.56 (↑0.45)	94.33 (↑0.72)	97.72	94.51	98.14 (↑0.42)	95.13 (↑0.62)	42.529	7.832
ResNet50	97.21	93.43	97.75 (↑0.54)	93.70 (↑0.27)	97.20	94.43	97.41 (↑0.21)	94.69 (↑0.26)	23.537	4.109
DenseNet121	95.68	91.63	96.58 (↑0.90)	91.99 (↑0.36)	95.68	92.63	96.58 (↑0.90)	93.02 (↑0.39)	6.968	2.865
MobileNetV3Large	97.66	93.43	98.20 (↑0.54)	93.52 (↑0.09)	96.46	94.43	97.18 (↑0.72)	94.52 (↑0.09)	5.450	0.225
EfficientNet	97.75	93.88	98.11 (↑0.36)	93.97 (↑0.09)	96.65	94.78	97.11 (↑0.46)	94.97 (↑0.19)	4.025	0.398
MobileNetV3Small	97.03	91.72	97.21 (↑0.18)	92.35 (↑0.63)	96.01	92.62	96.21 (↑0.20)	93.34 (↑0.72)	2.123	0.059
ShuffleNetV2	96.58	91.63	96.76 (↑0.18)	91.99 (↑0.36)	95.37	92.62	95.76 (↑0.39)	92.79 (↑0.17)	1.268	0.148
SqueezeNet	94.15	90.37	94.33 (↑0.18)	90.45 (↑0.08)	94.35	91.37	94.53 (↑0.18)	91.75 (↑0.38)	0.743	0.738

$T_{c}$ and $T_{s}$ represent the disease category recognition and severity estimation tasks in single-task models, respectively. $T_{hc}$ and $T_{hs}$ represent the corresponding tasks in multi-task models. The symbol ↑ symbol indicates Accuracy or F1-score improvement from the single-task baseline.

### 3.2 Results

#### 3.2.1 Performance comparison

In this section, we report the results from two experimental settings. The first setting, referred to as unified MTDL, employs the same network architecture for teacher and student modules. This setting aims to verify the effectiveness of the multi-stage distillation architecture proposed in this paper. The second setting, termed heterogeneous MTDL, involves using lightweight network architectures for all student models within the MTDL framework. This setting is designed to demonstrate the advantages of the proposed architecture in achieving a balance between performance and efficiency. As a reference, **Table 2** lists the baseline results of the initial two single tasks $T_{c}$ and $T_{s}$ , as well as the multi-task model $T_{h}$ , where $T_{hc}$ and $T_{hs}$ correspond to the results of $T_{h}$ for disease classification and severity estimation tasks, respectively. The results in **Table 2** demonstrate that the multi-task learning approach effectively enhances performance across various network architectures.

**Figure 4:**
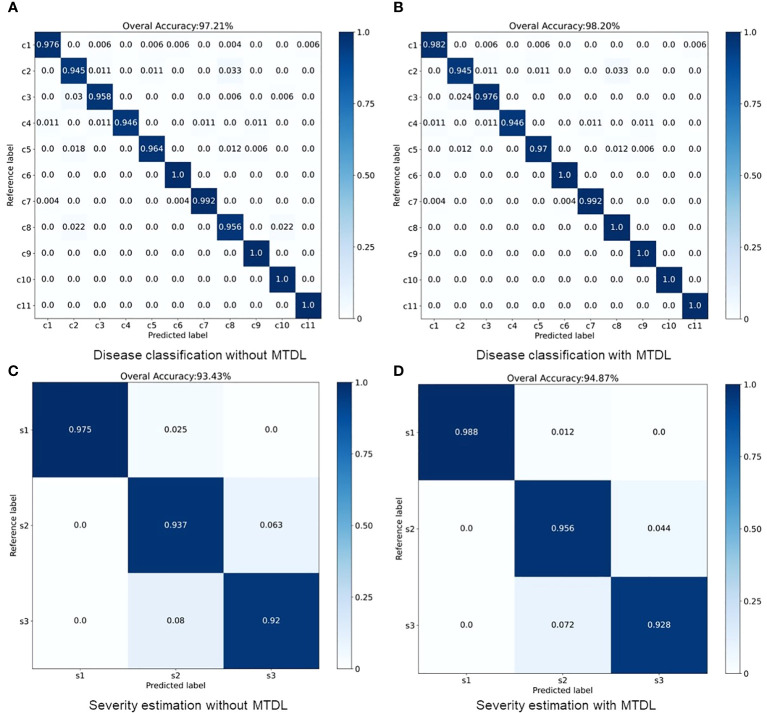
Performance improvement through multi-stage distillation in MTDL. **(A)** Disease classification without MTDL, **(B)** Disease classification with MTDL, **(C)** Severity estimation without MTDL, **(D)** Severity estimation with MTDL.

The results for MTDL with a unified architecture are presented in **Table 3**. We can observe that all models show improvement when using MTDL for knowledge learning. This indicates that the MTDL framework effectively leverages the staged learning of knowledge and the complementarity between different tasks. In terms of specific models, ResNet101 achieves the highest performance in both tasks under the MTDL setting, with Accuracy scores of 98.92% for $T_{c}$ and 95.32% for $T_{s}$ , respectively. The corresponding F1-scores are 98.78% and 96.32%, respectively. These results can be attributed to both the ResNet101’s powerful feature extraction capabilities and MTDL’s effective multi-task learning strategy. On the other hand, SqueezeNet shows significant improvement with an increase of 1.08% and 2.53% in Accuracy of $T_{c}$ and $T_{s}$ respectively, and an increase of 0.68% and 2.26% in F1-scoref or each task. This suggests that the MTDL allows the lightweight model to learn more robust and comprehensive features. Furthermore, **Table 3** also provides a comparison between the MTDL, MTDL-PTF, and MTDL-TF methods across various architectures. The results indicate that while the overall performance of MTDL-PTF and MTDL-TF decreases when the dependence on the teacher model is reduced, the introduction of a virtual teacher model significantly improves the accuracy of both methods compared to the original multitask learning. This indeed validates the effectiveness of the decoupled teacher-free knowledge distillation approach that we proposed. We also display the confusion matrices for results using ResNet50 as the backbone. As shown in **Figure 4**, it is evident that our proposed MTDL method either maintains or improves performance across all individual classes for both disease classification and severity estimation tasks. This demonstrates MTDL’s ability to achieve a balanced enhancement in both overall performance and category-specific outcomes.

**Table 3:** Performance of MTDL and its variants in a unified architecture.

Methods (Accuracy)	MTDL		MTDL-PTF		MTDL-TF	
	$T_{hc}^{\prime }$	$T_{hs}^{\prime }$	$T_{hc}^{v}$	$T_{hs}^{v}$	$T_{hc}^{v}$	$T_{hs}^{v}$
VGG16	97.75 (↑0.99)	94.15 (↑0.72)	97.48 (↑0.72)	94.24 (↑0.81)	97.12 (↑0.36)	93.70 (↑0.27)
ResNet101	98.92 (↑0.36)	95.32 (↑0.99)	98.65 (↑0.09)	94.87 (↑0.54)	98.65 (↑0.09)	94.78 (↑0.45)
ResNet50	98.20 (↑0.45)	94.87 (↑1.17)	98.11 (↑0.36)	94.60 (↑0.90)	97.93 (↑0.18)	94.34 (↑0.64)
DenseNet121	97.30 (↑0.72)	93.79 (↑1.80)	97.30 (↑0.72)	93.79 (↑1.80)	97.30 (↑0.72)	92.35 (↑0.36)
Average Improvement	↑0.63	↑1.17	↑0.47	↑1.01	↑0.34	↑0.43
MobileNetV3Large	98.74 (↑0.54)	94.60 (↑1.08)	98.65 (↑0.45)	94.24 (↑0.72)	98.56 (↑0.36)	93.97 (↑0.45)
EfficientNet	98.74 (↑0.63)	94.78 (↑0.81)	98.47 (↑0.36)	94.33 (↑0.36)	98.56 (↑0.45)	94.24 (↑0.27)
MobileNetV3Small	97.48 (↑0.27)	93.16 (↑0.81)	97.84 (↑0.63)	93.16 (↑0.81)	97.30 (↑0.09)	92.53 (↑0.18)
ShuffleNetV2	97.21 (↑0.45)	93.52 (↑1.53)	97.21 (↑0.45)	93.70 (↑1.71)	96.94 (↑0.18)	93.07 (↑1.08)
SqueezeNet	95.41 (↑1.08)	92.98 (↑2.53)	96.40 (↑2.07)	93.07 (↑2.62)	95.14 (↑0.81)	91.63 (↑1.18)
Average Improvement	↑0.59	↑1.35	↑0.79	↑1.24	↑0.38	↑0.63

Methods (F1-Score)	MTDL		MTDL-PTF		MTDL-TF	
	$T_{hc}^{\prime }$	$T_{hs}^{\prime }$	$T_{hc}^{v}$	$T_{hs}^{v}$	$T_{hc}^{v}$	$T_{hs}^{v}$
VGG16	97.85 (↑1.03)	95.15 (↑0.62)	97.47 (↑0.65)	95.24 (↑0.41)	96.96 (↑0.14)	94.77 (↑0.24)
ResNet101	98.78 (↑0.64)	96.32 (↑1.19)	98.46 (↑0.32)	95.86 (↑0.56)	98.49 (↑0.35)	95.68 (↑0.38)
ResNet50	97.52 (↑0.32)	95.87 (↑1.44)	98.11 (↑0.70)	95.58 (↑0.89)	97.59 (↑0.18)	95.24 (↑0.55)
DenseNet121	97.11 (↑0.53)	94.80 (↑1.78)	97.11 (↑0.53)	94.60 (↑1.58)	97.03 (↑0.45)	93.34 (↑0.32)
Average Improvement	↑0.63	↑1.26	↑0.55	↑0.86	↑0.28	↑0.37
MobileNetV3Large	97.65 (↑0.47)	95.60 (↑1.08)	97.41 (↑0.23)	95.24 (↑0.72)	97.25 (↑0.07)	94.56 (↑0.04)
EfficientNet	97.95 (↑0.84)	95.78 (↑0.81)	97.52 (↑0.41)	95.33 (↑0.36)	97.36 (↑0.25)	95.24 (↑0.27)
MobileNetV3Small	97.41 (↑1.20)	94.16 (↑0.82)	97.28 (↑1.07)	94.16 (↑0.82)	97.14 (↑0.93)	93.36(↑0.02)
ShuffleNetV2	97.01 (↑1.25)	94.52 (↑1.73)	97.01 (↑1.25)	94.60 (↑1.81)	96.74 (↑0.98)	94.27 (↑1.45)
SqueezeNet	95.21 (↑0.68)	94.01 (↑2.26)	96.52 (↑1.99)	94.27 (↑2.52)	94.97 (↑0.81)	92.63 (↑0.88)
Average Improvement	↑0.89	↑1.34	↑0.99	↑0.76	↑0.61	↑0.53

$T_{hc}^{\prime }$ and $T_{hs}^{\prime }$ represent MTDL’s performance, while $T_{hc}^{v}$ and $T_{hs}^{v}$ are for MTDL-PTF and MTDL-TF with a virtual teacher. The ↑ symbol indicates Accuracy and F1-score improvement, referencing the multi-task baseline from **Table 2**.

Furthermore, to investigate the impact of using teacher and student models with different architectures on the performance of the MTDL framework, we employ complex models like DenseNet121 for the teacher and lightweight models such as EfficientNet for the student. The results presented in **Table 4** substantiate the effectiveness of this heterogeneous MTDL approach. For instance, when using ResNet101 as the teacher model, the SqueezeNet student model shows an improvement of 1.95% and 3.07% in $T_{c}$ and $T_{s}$ respectively, which are higher than the result obtained under the unified architecture MTDL setting. These results suggest that a more powerful teacher model enriches the student model’s learning.

**Table 4:** Performance evaluation of MTDL under a heterogeneous setting.

Methods (Accuracy)		MTDL		Methods (Accuracy)		MTDL	
Teacher	Student	$T_{hc}^{\prime }$	$T_{hs}^{\prime }$	Teacher	Student	$T_{hc}^{\prime }$	$T_{hs}^{\prime }$
VGG16	MobileNetV3Large	98.74 (↑0.54)	94.51 (↑0.99)	ResNet50	MobileNetV3Large	98.92 (↑0.72)	94.42 (↑0.90)
	EfficientNet	98.47 (↑0.36)	94.54 (↑0.57)		EfficientNet	98.74 (↑0.63)	94.51 (↑0.54)
	MobileNetV3Small	97.48 (↑0.27)	93.52 (↑1.17)		MobileNetV3Small	97.66 (↑0.45)	94.15 (↑1.80)
	ShuffleNetV2	97.57 (↑0.81)	93.07 (↑1.08)		ShuffleNetV2	97.66 (↑0.90)	93.07 (↑1.08)
	SqueezeNet	95.95 (↑1.62)	92.62 (↑2.17)		SqueezeNet	96.04 (↑1.71)	92.98 (↑2.53)
Average Improvement		↑0.72	↑1.20	Average Improvement		↑0.88	↑1.37
ResNet101	MobileNetV3Large	98.92 (↑0.72)	95.05 (↑1.53)	DenseNet121	MobileNetV3Large	98.38 (↑0.18)	94.51 (↑0.99)
	EfficientNet	98.79 (↑0.68)	95.13 (↑1.16)		EfficientNet	98.47 (↑0.36)	94.87 (↑0.90)
	MobileNetV3Small	97.93 (↑0.72)	94.24 (↑1.89)		MobileNetV3Small	97.87 (↑0.66)	93.34 (↑0.99)
	ShuffleNetV2	98.02 (↑1.26)	93.97 (↑1.98)		ShuffleNetV2	97.48 (↑0.72)	93.79 (↑1.80)
	SqueezeNet	96.28 (↑1.95)	93.52 (↑3.07)		SqueezeNet	96.17 (↑1.84)	92.80 (↑2.35)
Average Improvement		↑1.07	↑1.93	Average Improvement		↑0.75	↑1.41

Methods (F1-Score)		MTDL		Methods (F1-score)		MTDL	
Teacher	Student	$T_{hc}^{\prime }$	$T_{hs}^{\prime }$	Teacher	Student	$T_{hc}^{\prime }$	$T_{hs}^{\prime }$
VGG16	MobileNetV3Large	98.54 (↑1.36)	95.24 (↑0.72)	ResNet50	MobileNetV3Large	98.72 (↑1.54)	95.62 (↑1.10)
	EfficientNet	97.98 (↑0.80)	95.36 (↑0.39)		EfficientNet	98.46 (↑1.35)	95.51 (↑0.54)
	MobileNetV3Small	97.46 (↑1.25)	94.52 (↑1.18)		MobileNetV3Small	97.66 (↑1.45)	94.10 (↑0.76)
	ShuffleNetV2	97.27 (↑1.51)	94.29 (↑1.50)		ShuffleNetV2	97.66 (↑1.45)	93.98 (↑1.19)
	SqueezeNet	95.76 (↑1.23)	93.42 (↑1.67)		SqueezeNet	96.04 (↑1.51)	93.67 (↑1.92)
Average Improvement		↑0.83	↑1.09	Average Improvement		↑1.46	↑1.10
ResNet101	MobileNetV3Large	98.62 (↑1.44)	95.85 (↑1.33)	DenseNet121	MobileNetV3Large	98.38 (↑1.20)	94.97 (↑0.45)
	EfficientNet	98.54 (↑1.43)	96.03 (↑1.06)		EfficientNet	98.27 (↑1.16)	95.62 (↑0.65)
	MobileNetV3Small	97.72 (↑1.51)	94.94 (↑1.60)		MobileNetV3Small	97.87 (↑1.66)	94.34 (↑1.00)
	ShuffleNetV2	98.22 (↑2.46)	94.87 (↑2.08)		ShuffleNetV2	97.28 (↑1.52)	94.09 (↑1.30)
	SqueezeNet	96.28 (↑1.75)	93.52 (↑1.77)		SqueezeNet	96.17 (↑1.64)	93.70 (↑1.95)
Average Improvement		↑1.72	↑1.57	Average Improvement		↑1.44	↑1.07

The ↑ symbol indicates an improvement in Accuracy and F1-score, as compared to the results listed in **Table 2**, where both teacher and student models use a unified lightweight network for multi-task learning.

Finally, to ensure the effectiveness of our proposed method, we conduct a comprehensive comparison with four well-established approaches in the field to validate its performance:

(a) Dual-stream hierarchical bilinear pooling (DHBP) (Wang et al., 2022): As a multi-task method initially developed for crops and diseases classification, we adapt DHBP for both disease classification and severity prediction tasks. This comparison allows us to evaluate the performance of our MTDL approach against a specialized multi-task learning method within the same domain.
(b) Traditional knowledge distillation (KD) (Ghofrani and Toroghi, 2022) and decouple knowledge distillation (DKD) (Zhao et al., 2022): These two methods represent the knowledge distillation category. We apply KD and its enhanced version, DKD, to our disease recognition and severity estimation tasks, providing a direct comparison with standard and advanced distillation techniques.
(c) Attention transfer (AT) (Komodakis and Zagoruyko, 2016): Differing from KD and DKD that focus on distilling knowledge through predicted outcomes, AT utilizes attention maps to transfer knowledge between the teacher and student models. Including AT in our comparison allows us to assess the efficacy of a distinct transfer learning approach.

To ensure fair comparisons among KD, DKD, AT, and MTDL, we consistently used ResNet-101 as the teacher and MobileNetV3Small as the student model. This approach enables a reliable assessment of knowledge distillation efficacy. Additionally, we present MTDL results using ResNet-101 as both teacher and student, aligning with DHBP’s backbone, to effectively demonstrate its multi-tasking capabilities.

The results are shown in **Table 5**. In our experiments, MTDL with ResNet-101 as both teacher and student models achieve the best results, outperforming DHBP in disease classification by 0.53% in Accuracy and 0.29% in F1-score, and in severity prediction by 0.86% in Accuracy and 1.08% in F1-score. These improvements validate MTDL’s phased multi-task learning approach. Moreover, when compared under the same teacher-student model setup with other distillation methods (KD, DKD, AT), MTDL excelled, particularly surpassing DKD by 0.37% in Accuracy and 0.16% in F1-score for disease classification, and by 0.62% in Accuracy and 0.38% in F1-score for severity prediction. This indicates the effectiveness of MTDL’s proposed mutual distillation learning between teachers and students.

**Table 5:** Comparative performance analysis of MTDL with other distillation-based and multi-task learning methods for disease classification and severity prediction.

Methods	Teacher	Student	Disease	Classification	Severity	Prediction
			Accuracy	F1-score	Accuracy	F1-score
DHBP (Wang et al., 2022)	ResNet101		98.39	98.49	94.46	95.24
KD (Ghofrani and Toroghi, 2022)	ResNet101	MobileNetV3Small	97.30	97.28	93.16	93.96
DKD Zhao et al. (2022)	ResNet101	MobileNetV3Small	97.56	97.56	93.62	94.56
AT Komodakis and Zagoruyko (2016)	ResNet101	MobileNetV3Small	97.39	97.46	93.28	94.09
MTDL	ResNet101 ResNet101	MobileNetV3Small ResNet101	97.93 98.92	97.72 98.78	94.24 95.32	94.94 96.32

#### 3.2.2 Significance analysis

In this subsection, we conduct a Wilcoxon Signed-Rank Test (Corder and Foreman, 2014) to evaluate the significance of the performance improvements across all CNN architectures. We provide the detailed significance analysis corresponding to the results originally presented in **Tables 3** and **4** in the following **Table 6** and **7**. In **Table 6**, we present a comparison of the performance of our MTDL model and its variants against several baseline CNN architectures. This table focuses on scenarios within our MTDL framework where both the teacher and student models utilize identical architecture. The results from this table demonstrate statistically significant improvements across all comparisons in both disease classification and severity prediction tasks. The p-values obtained are consistently well below the 0.05 threshold, indicating robust enhancements attributed to our MTDL approach. Similarly, **Table 7** showcases the results in a heterogeneous setting, where the MTDL model employs a more complex architecture as the teacher model and a lightweight network as the student model. In these comparisons, the results again confirm significant improvements across all evaluated aspects.

**Table 6:** Wilcoxon Signed-Rank Test results for MTDL variants’ Accuracy in a unified architecture.

Task	Model	vs VGG16	vs ResNet101	vs ResNet50	vs DenseNet121	
Disease Classification	MTDL	1.953 × 10−^3^	1.367 × 10^−2^	1.953 × 10^−3^	1.172 × 10^−2^	
	MTDL-PTF	1.953 × 10^−3^	1.065 × 10^−2^	1.953 × 10^−3^	1.953 × 10^−3^	
	MTDL-TF	1.953 × 10^−3^	2.066 × 10−^2^	4.980 × 10^−2^	1.953 × 10^−3^	
Severity Prediction	MTDL	1.953 × 10^−3^	1.953 × 10^−3^	1.953 × 10^−3^	1.953 × 10^−3^	
	MTDL-PTF	1.953 × 10^−3^	1.953 × 10^−3^	1.953 × 10^−3^	1.953 × 10^−3^	
	MTDL-TF	1.953 × 10^−3^	1.953 × 10^−3^	1.953 × 10^−3^	1.953 × 10^−3^	
Task	Model	vs MobileNetV3Large	vs EfficientNet	vs MobileNetV3Small	vs ShuffleNetV2	vs SqueezeNet
Disease Classification	MTDL	1.151 × 10^−2^	1.953 × 10^−3^	3.906 × 10^−3^	1.953 × 10^−3^	1.953 × 10^−3^
	MTDL-PTF	1.172 × 10^−1^	1.079 × 10^−2^	1.953 × 10^−3^	1.953 × 10^−3^	1.953 × 10^−3^
	MTDL-TF	4.206 × 10^−2^	1.065 × 10^−2^	3.906 × 10^−3^	1.953 × 10^−3^	1.953 × 10^−3^
Severity Prediction	MTDL	1.953 × 10^−3^	1.953 × 10^−3^	1.953 × 10^−3^	1.953 × 10^−3^	1.953 × 10^−3^
	MTDL-PTF	1.953 × 10^−3^	3.906 × 10^−3^	1.953 × 10^−3^	1.953 × 10^−3^	1.953 × 10^−3^
	MTDL-TF	1.278 × 10^−2^	2.734 × 10−2	1.079 × 10^−2^	1.953 × 10^−3^	1.953 × 10^−3^

**Table 7:** Wilcoxon Signed-Rank Test results for MTDL variants’ Accuracy under heterogeneous settings (‘()’ indicate teacher models).

Task	Model	vs MobileNetV3Large	vs EfficientNet	vs MobileNetV3Small	vs ShuffleNetV2	vs SqueezeNet
Disease Classification	MTDL (VGG16)	7.632 ×10^−3^	1.162 ×10^−2^	1.953 ×10^−3^	1.953 ×10^−3^	1.953 ×10^−3^
	MTDL (ResNet101)	1.953 ×10^−3^	1.953 ×10^−3^	1.953 ×10^−3^	1.953 ×10^−3^	1.953 ×10^−3^
	MTDL (ResNet50)	1.953 ×10^−3^	1.953 ×10^−3^	3.906 ×10^−3^	1.953 ×10^−3^	1.953 ×10^−3^
	MTDL (DenseNet121)	1.953 ×10^−3^	1.953 ×10^−3^	1.953 ×10^−3^	1.953 ×10^−3^	1.953 ×10^−3^
Severity Prediction	MTDL (VGG16)	1.953 ×10^−3^	1.953 ×10^−3^	1.953 ×10^−3^	1.953 ×10^−3^	1.953 ×10^−3^
	MTDL (ResNet101)	1.953 ×10^−3^	1.953 ×10^−3^	1.953 ×10^−3^	1.953 ×10^−3^	1.953 ×10^−3^
	MTDL (ResNet50)	1.953 ×10^−3^	1.953 ×10^−3^	1.953 ×10^−3^	1.953 ×10^−3^	1.953 ×10^−3^
	MTDL (DenseNet121)	1.953 ×10^−3^	1.953 ×10^−3^	1.953 ×10^−3^	1.953 ×10^−3^	1.953 ×10^−3^

In addition, we also perform the significance of the results in comparison with other multi-task and distillation learning methods. with the results recorded in **Table 8**. It can be seen that in most cases, the MTDL framework shows statistically significant differences when compared with methods like DHBP, KD, DKD, and AT, with p-values well beneath the 0.05 significance threshold. However, there is one exception to note: in the case of MTDL (ResNet101-MobileNetV3Small) vs DHBP for severity prediction, the p-value is slightly above the conventional threshold for significance. This exception likely stems from MTDL employing lightweight MobileNetV3Small as the distillation target, whereas DHBP uses the more substantial ResNet101 as its base model.

**Table 8:** Results of the Wilcoxon Signed-Rank Test for MTDL and its variants versus other methods (The first in ‘()’ is the teacher model and the second is the student model).

Task	Model	vs DHBP	vs KD	vs DKD	vs AT
Disease Classification	MTDL (ResNet101-ResNet101)	1.507×10^−2^	1.953×10^−3^	1.953×10^−3^	1.953×10^−3^
	MTDL (ResNet101-MobileNetV3Small)	1.953×10^−3^	1.953×10^−3^	1.953×10^−3^	1.953×10^−3^
Severity Prediction	MTDL (ResNet101-ResNet101)	1.953×10^−3^	1.953×10^−3^	1.953×10^−3^	1.953×10−3
	MTDL (ResNet101-MobileNetV3Small)	9.219×10−2	1.953×10^−3^	1.953×10−3	1.953×10−3

### 3.3 Discussion

#### 3.3.1 The effectiveness of multi-stage distillation learning

**Figure 5:**
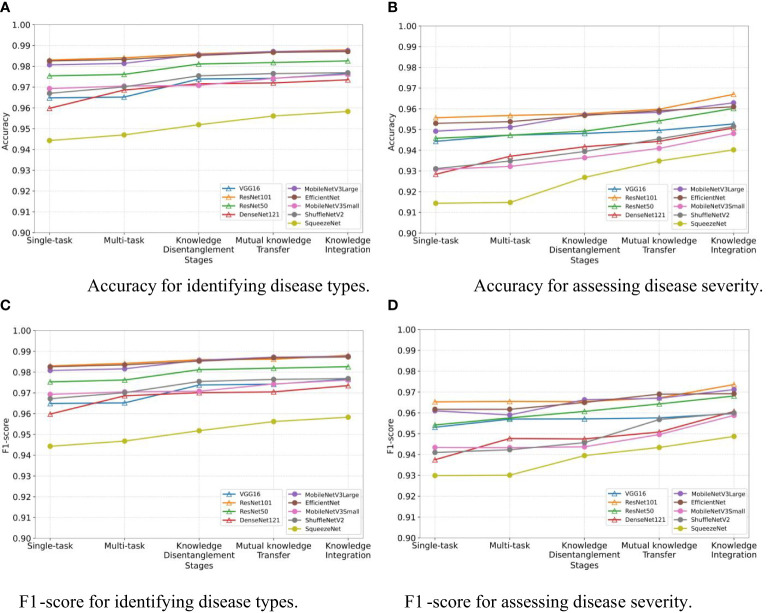
Performance improvement through multi-stage distillation in MTDL. **(A)** Accuracy for identifying disease types, **(B)** Accuracy for assessing disease severity, **(C)** F1-score for identifying disease types, **(D)** F1-score for assessing disease severity.

We assess the effectiveness of the three stages in our MTDL framework: knowledge disentanglement, mutual knowledge transfer, and knowledge integration. To do so, we employ single-task and multi-task models as our baselines and incorporate the results obtained after each stage of learning. As illustrated in **Figure 5**, the results in terms of Accuracy and F1-score align with our expectations. The results clearly demonstrate that each stage of learning contributes to the final performance improvement, thereby validating the effectiveness of staged distillation in the MTDL framework.

#### 3.3.2 Trade-off between performance and efficiency

**Figure 6:**
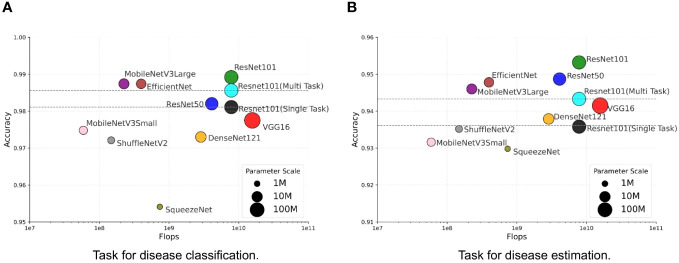
Trade-off between performance and efficiency. **(A)** Task for disease classification, **(B)** Task for disease estimation.

We investigate the balance between performance and efficiency within the context of our MTDL framework. Performance is measured by Accuracy, while efficiency is represented by the number of parameters and floating-point operations (FLOPs). We use the single-task ResNet101 model and the multi-task ResNet101 model as baselines due to their superior performance across all single-task and multi-task models, as shown in **Table 3**. The results are presented in **Figure 6**, and the size of each model’s marker in the figure represents the number of parameters used by the model.

It can be observed that there is a similar trend in both task of disease classification (**Figure 6A**) and disease severity estimation (**Figure 6B**). Our MTDL-enhanced ResNet101 notably surpasses the single-task baseline with an Accuracy improvement of 0.81% for disease classification and 1.71% for severity estimation, and it outperforms the multi-task baseline with 0.36% and 0.99% improvements respectively. When using MobileNetV3Large as the MTDL-optimized model, we achieved significant performance gains with reduced parameter count and FLOPs, while still enhancing Accuracy over both baselines. For example, the MobileNetV3Large model, enhanced by our MTDL framework, outperforms the ResNet101 baseline by 0.63% and 1.44% in the two tasks, respectively. Remarkably, this is achieved with only 12.81% of the parameters (5.450M vs. 42.529M) and 2.87% of the FLOPs (0.225G vs. 7.832G). These findings highlight the MTDL framework’s capability to improve performance significantly while maintaining computational efficiency, thereby reinforcing its advantage over conventional models.

Therefore, we need to select the appropriate distillation model for each specific scenario. The choice depends on balancing computational resources and performance. Typically, complex teachers like ResNet101 outperform compact students such as MobileNet, owing to deeper architectures. MTDL promotes mutual learning between teachers and students, simultaneously enhancing both models. With abundant resources, an MTDL-optimized teacher offers substantial performance gains. In contrast, for limited-resource scenarios like mobile inference, MTDL can distill a lightweight yet performant student model. Additionally, the teacher-free MTDL-TF variant reduces dependency on complex teachers, offering an alternative when resources are constrained.

#### 3.3.3 Visual analysis for multi-task learning

**Figure 7:**
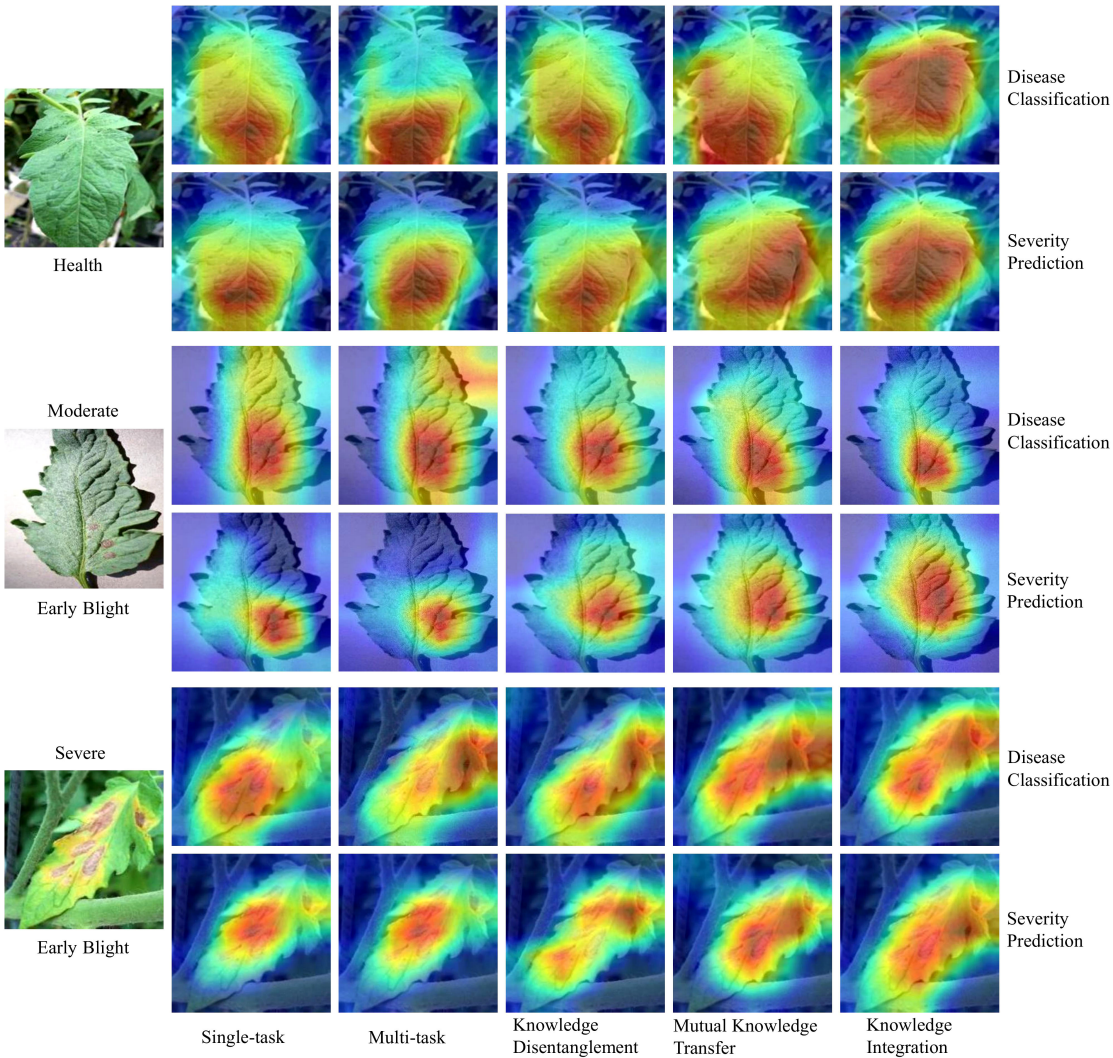
Visual analysis of attention shifts in MTDL framework across severity levels.

In this section, we use Grad-CAM (Selvaraju et al., 2017) for visual analysis to gain deeper insights into the learning process of our MTDL framework. We examine three severity levels of Early Blight: healthy, general, and severe. Visualizations for single-task and multi-task models, as well as for each stage of MTDL learning, are provided. **Figure 7** shows that the model’s attention shifts toward task-relevant areas as it learns. For healthy leaves, the MTDL-enhanced model more precisely identifies the leaf as a whole, aligning with human visual systems. For leaves at a general severity level, the model focuses on localized disease spots for classification but expands its attention to surrounding regions for severity estimation. In cases of severe disease levels, the disease spots typically exhibit a widespread distribution across the leaf area. The knowledge integration model, in its pursuit to accurately recognize both the disease type and severity, tends to produce a Grad-CAM sensitivity map covering the entire leaf area. This comprehensive coverage contrasts with the single-task model, which primarily focuses on localized diseased regions, and the multi-task model, which, although it expands the area of interest, does not distribute sensitivity intensity as effectively. Moreover, the distribution of sensitivity intensity in the knowledge integration model offers a more realistic representation of the disease’s extensive impact, thereby enhancing the model’s explanatory power for Severe Early Blight. This analysis highlights the MTDL framework’s adaptability in shifting its focus based on the task and severity, thereby improving performance and interpretability.

#### 3.3.4 Parameter sensitivity analysis

**Figure 8:**
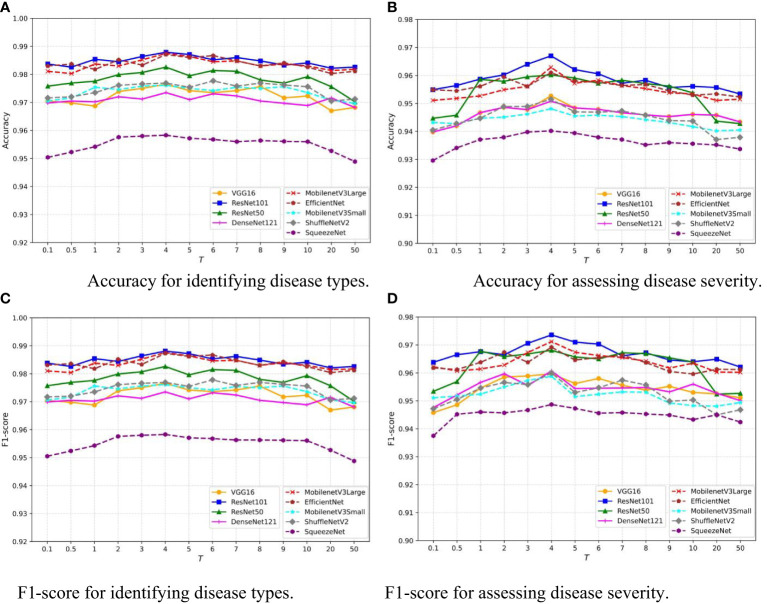
Sensitivity analysis of temperature hyperparameter *T* in MTDL framework. **(A)** Accuracy for identifying disease types, **(B)** Accuracy for assessing disease severity, **(C)** F1-score for identifying disease types, **(D)** F1-score for assessing disease severity.

The temperature parameter *T* adjusts the softmax output in the neural network, smoothing the probability distribution and revealing more nuanced information about the model’s predictions. This is crucial for knowledge distillation, where it aids in transferring detailed information from a teacher to a student model. This concept is introduced and utilized in Equation 1. To assess the sensitivity of our model to *T*, we vary *T* within the interval [0.1,50] and record the Accuracy of the disease classification and severity estimation tasks for each value. The results of nine common network architectures are shown in **Figure 8**. Despite the differences in architecture, a similar trend is observed: as *T* increases, the model’s performance improves, but rapidly declines when *T* exceeds 10. Notably, the model’s performance remains relatively stable for *T* within the interval [3,8]. This indicates that our model is robust to the choice of *T* within this range, providing flexibility in practical applications.

**Figure 9:**
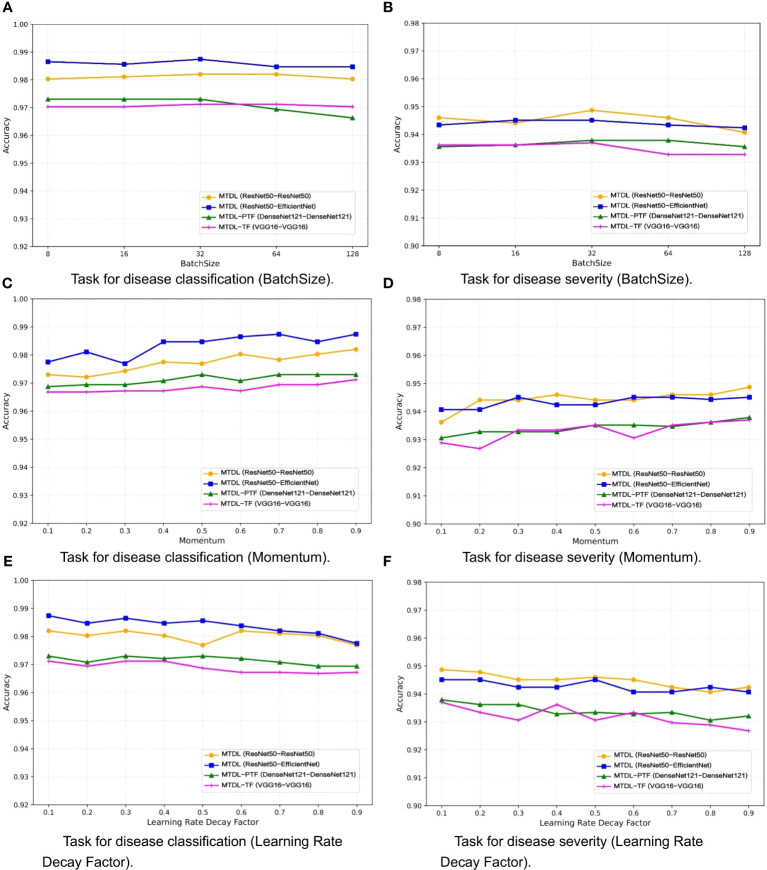
Effect of different parameters on model performance. **(A)** Task for disease classification (BatchSize), **(B)** Task for disease severity (BatchSize), **(C)** Task for disease classification (Momentum), **(D)** Task for disease severity (Momentum), **(E)** Task for disease classification (Learning Rate Decay Factor), **(F)** Task for disease severity (Learning Rate Decay Factor).

One the other hand, the selection of a batch size of 32, momentum of 0.9, and learning rate decay factor of 0.1 was guided by a combination of empirical conventions and experimental validation aimed at striking a balance between computational efficiency and model performance. To validate the impact of different parameter settings on performance, we analyzed MTDL and its variants on the validation set for varying batch sizes (**Figures 9A, B**), momentum (**Figures 9C, D**), and learning rate decay factors (**Figures 9E, F**), detailing their effects on Accuracy. We can see that Accuracy remains relatively stable across batch sizes that varies (8, 16, 32, 64, 128), with the optimal average Accuracy achieved at 32. This is likely because a moderate batch size balances gradient estimation Accuracy and the beneficial noise of stochasticity, optimizing learning. As momentum increases from 0.1 to 0.9, Accuracy generally improves. A higher momentum, like 0.9, effectively uses past gradients to accelerate convergence and navigate through local minima, leading to better performance compared to a lower setting like 0.1. Moreover, increasing decay factors tend to lower Accuracy, potentially due to a swift reduction in the learning rate and premature convergence. An optimal decay factor is one that slowly decreases the learning rate, facilitating precise adjustments as the model converges to the best solution.

## 4 Conclusion

In this work, we present the multi-task distillation learning (MTDL) framework, a specialized solution for diagnosing tomato diseases. The framework comprises three key stages: knowledge disentanglement, mutual knowledge transfer, and knowledge integration. Using this staged learning approach, we leverage the complementary aspects of different tasks to enhance performance across various network architectures. Moreover, our framework adeptly balances performance with efficiency, underlining its potential for practical applications. Although MTDL enhances traditional knowledge distillation with bidirectional knowledge transfer between teacher and student models, it extends training time due to a progressive, multi-stage learning approach. To mitigate this, we introduce MTDL-PTF and MTDL-TF variants for efficiency, though they may slightly underperform compared to the original MTDL.

Furthermore, our current framework has some limitations. First, although the framework is designed for outdoor environments, it has stringent requirements for the subject being photographed, focusing mainly on recognizing single subjects in images. Second, the severity level classification is relatively basic, encompassing only three levels, including a healthy state. In future work, we plan to integrate object localization techniques into the distillation process to facilitate the identification of multiple leaves in images. Additionally, we aim to refine the classification of disease severity levels, focusing especially on the early detection of diseases. These planned enhancements will contribute to the development of more sophisticated and nuanced solutions in the field of tomato disease diagnosis, offering a robust framework for sustainable and intelligent agriculture.

## Data availability statement

The raw data supporting the conclusions of this article will be made available by the authors, without undue reservation.

## Author contributions

BL: Conceptualization, Methodology, Writing – original draft. SW: Data curation, Formal analysis, Software, Writing – review & editing. FZ: Funding acquisition, Methodology, Writing – review & editing. NG: Formal analysis, Validation, Writing – review & editing. HF: Data curation, Formal analysis, Validation, Writing – review & editing. WY: Project administration, Writing – review & editing.
